# Effects of Whole “Navelina” Orange Consumption on Glucose Homeostasis and Fructosamine Levels in Patients with Metabolic Dysfunction-Associated Steatotic Liver Disease (MASLD): A Randomized Controlled Trial

**DOI:** 10.3390/nu18213668

**Published:** 2026-11-05

**Authors:** Valentina De Nunzio, Davide Guido, Giuliano Pinto, Emanuela Aloisio Caruso, Miriam Cofano, Ilenia Saponara, Matteo Centonze, Sabrina De Tullio, Annalisa Ferro, Maria Grazia Refolo, Maria Notarnicola

**Affiliations:** 1 Laboratory of Nutritional Biochemistry, National Institute of Gastroenterology IRCCS “Saverio de Bellis”, 70013 Castellana Grotte, Italy; valentina.denunzio@irccsdebellis.it (V.D.N.); giuliano.pinto@irccsdebellis.it (G.P.); emanuela.caruso@irccsdebellis.it (E.A.C.); miriam.cofano@irccsdebellis.it (M.C.); ilenia.saponara@irccsdebellis.it (I.S.); matteo.centonze@irccsdebellis.it (M.C.); detulliosabrina02@gmail.com (S.D.T.); 2 Data Science Unit, National Institute of Gastroenterology IRCCS “Saverio de Bellis”, 70013 Castellana Grotte, Italy; davide.guido@irccsdebellis.it; 3 Laboratory of Clinical Pathology, National Institute of Gastroenterology IRCCS “Saverio de Bellis”, 70013 Castellana Grotte, Italy; annalisa.ferro@irccsdebellis.it (A.F.); maria.refolo@irccsdebellis.it (M.G.R.)

**Keywords:** MASLD, glucose metabolism, fructosamine, oranges, randomized clinical trial

## Abstract

**Background**: Metabolic dysfunction-associated steatotic liver disease (MASLD) is closely linked to insulin resistance and altered glucose and lipid metabolism. Fructosamine assays can provide complementary information regarding short-term glycemic exposure, making them particularly useful in the context of short-term nutritional interventions. This study evaluated the effects of whole “Navelina” orange consumption on glucose homeostasis in patients with MASLD. **Methods**: Sixty patients with MASLD were randomly assigned to an experimental or control arm. The experimental arm consumed 400 g/day of whole “Navelina” oranges for 4 weeks, while controls abstained from orange consumption. Fasting glucose, insulin, fructosamine, HOMA-IR, and QUICKI were assessed at baseline and follow-up. Generalized estimating equation models evaluated time-by-treatment interactions. Exploratory Spearman correlations assessed associations between changes in the lipid and glucose-related parameters. **Results**: Significant time–treatment interactions were observed for dietary sugars, vitamin A, and vitamin C. No significant interactions were observed for fasting glucose, insulin, HOMA-IR, or QUICKI. Fructosamine levels showed a non-significant trend toward a reduction (*p* = 0.078). In the experimental arm, changes in LDL levels were positively correlated with HOMA-IR and insulin levels, and inversely correlated with QUICKI. **Conclusions**: This study is one of the first clinical trials to evaluate the potential impact of whole orange consumption on glucose metabolism. However, these exploratory findings require confirmation through randomized studies conducted on larger samples and over longer periods.

## 1. Introduction

Metabolic and fatty liver disease (MASLD) is the most common chronic liver disease in the world and is closely associated with obesity, insulin resistance, type 2 diabetes, and dyslipidemia. MASLD reflects a systemic metabolic disorder in which abnormalities in glucose and lipid metabolism contribute to the development and progression of hepatic steatosis. Consequently, current guidelines recommend an integrated management of cardiometabolic risk factors, with lifestyle and dietary modifications as key components of treatment [1,2].

Dietary modification is a key component of MASLD management, as dietary composition can influence hepatic steatosis and metabolic health independently of weight loss [3]. Excessive intake of free sugars, particularly when consumed in energy-dense foods and sugar-sweetened beverages, may promote hepatic de novo lipogenesis and metabolic dysfunction [4]. However, these effects may depend on the food matrix and energy context, suggesting that sugars consumed in the form of fresh fruit may have different metabolic effects than added sugars and sugary beverages [3,5].

Citrus fruits and their derivatives have been of particular interest as potential components of dietary strategies for metabolic health, given their content of dietary fiber, vitamin C, and a variety of polyphenols, including flavonoids such as hesperidin. These compounds may exert complementary antioxidant and metabolic effects, potentially influencing glucose and lipid metabolism, insulin sensitivity, and oxidative stress [6,7].

Randomized clinical trials and recent meta-analyses have reported that hesperidin supplementation can improve glucose profiles by modifying some biomarkers of insulin sensitivity, including homeostatic model assessment for insulin resistance (HOMA-IR) and the quantitative insulin sensitivity check index (QUICKI), and by reducing fasting blood glucose in specific contexts [8,9]. Similarly, studies conducted on the consumption of orange juice have shown favorable changes in glucose, insulin, and indices of insulin resistance, supporting the hypothesis that the metabolic effects of citrus fruit consumption may depend not only on the individual bioactive compounds but also on their interaction [10,11,12].

In addition to fasting glucose and insulin measurements, the use of biomarkers capable of detecting short-term changes in glycemic exposure may be particularly relevant in nutritional interventions of limited duration [13]. Fructosamine reflects the glycation of circulating proteins and provides an estimate of average glycemic exposure over approximately the preceding 2–3 weeks. Thus, unlike glycated hemoglobin, which reflects a substantially longer period of glycemic exposure, fructosamine may provide complementary information when evaluating metabolic changes occurring during short-term interventions [13,14].

Despite its potential usefulness, evidence regarding fructosamine in patients with MASLD remains limited. Kamuzinzi et al. analyzed serum fructosamine by the severity of hepatic steatosis and found no significant differences between patients with mild, moderate, or severe steatosis [15]. However, this study was cross-sectional and focused on the diagnostic association between fructosamine and the severity of MASLD, rather than on its ability to detect metabolic changes following a dietary intervention [15].

Some evidence supports a potential link between glycation-related pathways and the metabolic phenotype of MASLD. Palma-Duran et al. reported higher levels of several advanced glycation biomarkers end-products and altered advanced glycation end-products (AGE)/receptor for AGEs (sRAGE)-related measures in normoglycemic individuals with MASLD compared with matched controls [16]. Importantly, several glycation-related markers showed associations with metabolic and hepatic parameters, including HOMA-IR, supporting the concept that altered glycation may be involved in the metabolic environment characterizing MASLD even in the absence of overt hyperglycemia. However, whether fructosamine is sensitive to short-term changes induced by a whole-food nutritional intervention in patients with MASLD remains poorly understood.

In this context, whole orange consumption represents an interesting nutritional model. Unlike orange juice or isolated citrus compounds, consumption of whole oranges provides sugars within a complex food matrix together with fiber and bioactive compounds. In our randomized clinical trial conducted in patients with MASLD, we previously demonstrated that a four weeks daily consumption of 400 g of “Navelina” whole oranges was associated with a significant reduction in the prevalence of hepatic steatosis, despite the absence of significant changes in body weight, body composition, fasting glucose or insulin [17]. These findings suggested that the effects of whole-orange consumption on hepatic health might occur independently of weight loss and without detectable changes in conventional fasting glycemic parameters.

More recently, we investigated serum lipid levels and lipidomic profiles in the same randomized clinical trial [18]. Although no significant treatment-by-time interaction was observed for the main lipidomic variables, the intervention group showed several associations between circulating fatty acids and high-density lipoprotein (HDL), including positive correlations of eicosapentaenoic acid (EPA) and *n*-3 polyunsaturated fatty acids (PUFAs) with HDL and inverse correlations of oleic acid, monounsaturated fatty acids (MUFAs) and the arachidonic acid (AA)/EPA ratio with HDL.

In line with our previous investigations focusing on hepatic steatosis and circulating lipid and lipidomic profiles, the present study provides a complementary analysis of the metabolic effects of a four-week intervention involving the consumption of 400 g/day of “Navelina” oranges in patients with MASLD, specifically addressing glucose homeostasis and short-term glycemic control. With particular emphasis on serum fructosamine as a marker of short-term glycemic exposure. Fasting glucose, insulin, HOMA-IR and QUICKI were evaluated as indicators of glucose metabolism and insulin sensitivity. In addition, given the close metabolic relationship between insulin resistance and lipid metabolism, we explored the associations between glucose-related parameters and circulating lipid biomarkers.

## 2. Materials and Methods

### 2.1. Participants

A total of 60 subjects (43 men, 71.67%), aged between 30 and 65 years, with a diagnosis of MASLD were enrolled at the National Institute of Gastroenterology IRCCS “S. de Bellis” between February 2023 and November 2023. Eligibility criteria included the presence of fatty liver disease, defined by a Controlled Attenuation Parameter (CAP) value > 275 dB/m, in combination with overweight (Body Mass Index, BMI > 25 kg/m^2^), type 2 diabetes mellitus, and/or metabolic syndrome, according to previously established criteria [17]. The study protocol was approved by the Human Studies Committee of the IRCCS Oncological Hospital—Giovanni Paolo II, Bari, Italy (Approval No. #184, 13 May 2022). Written informed consent was obtained from all participants in accordance with the principles of the Declaration of Helsinki. The clinical trial was registered on ClinicalTrials.gov under the identifier NCT05558592.

Participants were randomly allocated to a control arm or an experimental treatment arm according to a computer-generated randomization sequence based on non-unique, unordered numbers ranging from 1 to 2. Subjects allocated to the experimental arm were asked to consume 400 g of “Navelina” oranges daily for a period of 4 weeks. All participants received dietary advice, including recommendations to limit the intake of alcohol, caffeine, and foods nutritionally similar to citrus fruits. Participants assigned to the control arm were instructed to abstain from consuming oranges for the entire duration of the study.

Blood samples were collected from all participants, including those in the control arm, at baseline and following the 4-week intervention. To monitor dietary adherence, all subjects completed a self-reported adherence questionnaire and maintained a food diary during the week preceding the intervention and throughout the study period.

### 2.2. Blood Sampling and Biochemical Measurements

Venous blood samples were obtained from all participants following a 12-h overnight fast at baseline and after 4 weeks of intervention (follow-up). Samples were collected into tubes containing silica gel as a clotting activator and subsequently centrifuged at 3200 rpm for 10 min to obtain serum.

Serum concentrations of fasting blood glucose, fasting insulin, HOMA-IR test and fructosamine were measured at the Laboratory of Clinical Pathology of the Institute using Cobas 8000 automated analyzers (Roche Diagnostics S.p.A., Monza, Italy).

Additionally, the Quantitative Insulin Sensitivity Index (QUICKI), a mathematical formula used to estimate the body’s response to insulin from a single fasting blood sample, was calculated. The QUICKI is calculated using the following equation:

$$\mathrm{QUICKI}=\;\frac{1}{\left[\log\left(\mathrm{fasting}\;\mathrm{insulin}\right)+\;\log\left(\mathrm{fasting}\;\mathrm{blood}\;\mathrm{glucose}\right)\right]}$$

### 2.3. Dietary Assessment

Dietary intake was assessed using a 7-day food diary completed by all participants during the week preceding the intervention and throughout the 4-week study. Participants were asked to record all foods and beverages consumed, along with their portion sizes.

The food diaries were subsequently reviewed and analyzed using MetaDieta software (version 4.0.1 accessed on 16 September 2024) to quantify daily energy and nutrient intake. The following daily dietary parameters were assessed: total energy intake; intake of proteins, lipids, carbohydrates, starches, sugars, and total dietary fiber; intake of sodium, potassium, iron, calcium, and phosphorus; vitamins B1, B2, B3, A, C, and E; water intake; and dietary fatty acid composition, including saturated (SFAs), monounsaturated (MUFAs), and polyunsaturated (PUFAs) fatty acids.

### 2.4. Statistical Analysis

Continuous variables were reported as median with interquartile range (IQR) or as mean ± standard deviation, whereas categorical variables were presented as frequencies and percentages. At baseline, differences between treatment groups were initially assessed using the non-parametric Mann–Whitney test for continuous variables and the Chi-squared test or Fisher’s exact test for categorical variables, as appropriate.

#### 2.4.1. GEE Modelling

Generalized estimating equation (GEE) models [19,20] were used to assess differences over time between treatment groups regarding glycemic, biological, and clinical markers. Theoretically, GEE models are typically used to analyze correlated data, such as repeated measures over time or clustered data. They are marginal models (population-averaged) estimating the average effect of covariates on the response variable in the population, accounting for within-group correlation without explicitly modeling random effects. Accounting for this, GEE models are also considered for their robustness. In this study, they allowed the management of the intra-subject variability produced by two separate measurements carried out on the same patients (n = 30 × 2 = 60 observations per arm, but only 30 independents) [21]. For each outcome, a GEE model was defined including time, treatment group, and their interaction as primary predictors. Time was treated as a two-level categorical variable, with the baseline value as the reference category and the 4-week follow-up as the second level. Treatment was defined as a two-level factor, with the control arm as the reference category and the experimental treatment as the comparison group. The models’ time parameters (β_T_) were interpreted as average time changes in the outcomes between the two separate measurements (follow up − baseline measurements); the parameters associated with the treatment (β_TR_) were interpreted as average baseline differences between the two treatments (the control one represented the reference category). Finally, the interaction parameters (β_TxTR_) were interpreted as average time differences in the outcomes (from baseline) between treatments. Notably, the expected within-group changes were assessed by jointly considering the time parameter and the interaction, as required by the GEE model specification [19]. Moreover, estimates were adjusted for baseline values of total and HDL cholesterol, dietary lipids, and intakes of proteins and carbohydrates to manage imbalances across treatments by avoiding the risk of confounding. These potential confounders were selected because clinically relevant by accounting for the 20:1 sample size ratio (i.e., 20 statistical units per 1 covariate) [22] for the quantitative response variables. Hence, it is worth to clarifying out that the fructosamine has not been included in the set of the potential confounders in the GEE modelling because the sample size (in terms of measurements, i.e., n = 30 × 2 = 60 observations per arm = 120) did not allow to include a number of covariates bigger than 5 (added to the two main effects and treatment-by-time interaction).

It is worth pointing out that given the skewed distribution of continuous outcomes, Gamma GEE models with an identity link function were used where appropriate. An exchangeable working correlation structure was specified, and sandwich-based robust standard errors were adopted to account for potential model misspecification. In addition, Gaussian GEE models were applied to outcomes including null values. In general, the Gamma distribution is defined over positive values only (i.e., y > 0), whereas the Gaussian distribution has support over the entire real-number range (i.e., −Inf, +Inf).

The decision to employ both Gaussian and Gamma GEE models was driven by the specific distributional properties and support of the continuous outcomes analyzed in our study: Gaussian models are most appropriate for continuous outcomes that are approximately symmetrically distributed; Gamma distribution is inherently right-skewed. In addition, applying a Gaussian model to strictly positive variables can lead to biased estimates or predicted values that fall below zero, which is biologically implausible.

#### 2.4.2. Correlation Analysis

Finally, a Spearman correlation analysis was performed to investigate the association between fructosamine, insulin, HOMA-IR test, QUICKI test and low-density lipoprotein (LDL), after 4 weeks in the arm-stratified samples.

The Spearman correlation coefficient (r) is commonly used to evaluate the raw association between two variables in a non-parametric framework (i.e., lack of normality). The value of r covers the whole range of relationship strengths, from no relationship whatsoever (r = 0) to a perfect relationship (r = 1 or r = −1) [23]. According to Cohen’s guidelines, correlation coefficients of |0.1|, |0.3|, and |0.5| can be interpreted as indicating small, medium, and large effect sizes, respectively [24,25].

Wald tests and 95% confidence intervals were carried out to evaluate statistical significance on model parameters. Two-tailed *p*-values < 0.05 were considered significant. Calculations were carried out using the statistical software R (version 4.3.3) (R Core Team, 2024) and its packages geepack [19,26,27], Hmisc [28], pwr [29] and ggplot2 [30].

## 3. Results

Table 1 shows demographic data, anthropometric measurements, glucose metabolism and weekly dietary intake parameters at baseline. Adherence to the intervention was consistently high in both groups, with overall compliance reaching 96.8% in the experimental arm and 93.1% in the control arm.

Regarding the GEE modeling, Table 2 outlines the results in terms of coefficients, *p*-values, and 95% CIs. Sugars, vitamin A and vitamin C provided significant time–treatment interactions (β_TxTR_) across the modeling, by showing increasing differences in the experimental arm across time, in relation to the control one. In addition, the GEE analysis revealed a trend in the interaction term (β_TxTR_) towards a decrease for fructosamine (*p*-value = 0.078) and an increase for vitamin B1 and potassium (0.058 and 0.053, respectively).

Table 3 presents the results of the Spearman correlation analysis (r) regarding the changes (∆) (follow-up − baseline) in glucose metabolism and LDL for each arm. In the experimental arm, no correlation was observed for fructosamine, whereas LDL was significantly and positively correlated with the HOMA-IR test and insulin (r = 0.471, *p* = 0.009; r = 0.475, *p* = 0.008, respectively). A significant negative correlation was also found between LDL and the QUICKI test (r = −0.639, *p* < 0.001). In both arms, correlations were observed between insulin levels and the HOMA-IR and QUICKI indices. These associations can also be considered in light of how the two indices are calculated, as insulin is included among the variables used to determine them. Despite that, no significant correlations were observed in the control arm. These correlations do not establish a treatment-related metabolic mechanism.

To gain further insight into the findings obtained from the LDL correlation analyses, scatter plots including fitted regression lines, together with level plots, were produced (Figure 1, Figure 2, Figure 3 and Figure 4). These graphical representations describe the relationship between changes in LDL (∆) and the values of fructosamine, HOMA-IR, insulin, and QUICKI according to treatment group. Panels a and b display the individual patient observations as x–y scatter plots, whereas panels c and d provide level plots based on two-dimensional kernel density estimates, illustrating the concentration and distribution of the observations across the x–y space.

Within the experimental arm, a positive association was observed between HOMA-IR values and changes in LDL, indicating that higher HOMA-IR values tended to coincide with greater LDL levels (Figure 2). A comparable pattern emerged for insulin concentrations (Figure 3). In contrast, QUICKI values showed an inverse relationship with LDL changes following orange consumption (Figure 4)

These findings should, however, be interpreted with caution, as the correlation analyses were conducted for exploratory purposes. Consequently, they do not provide evidence of a causal treatment effect and should not be interpreted as demonstrating a specific underlying mechanism of action.

It is worth pointing out that the correlation analyses are exploratory in nature and should not be used to imply treatment effects or mechanism.

Finally, post hoc analysis was performed on the observed standardized mean differences in the Δ-changes in the fasting blood glucose and fructosamine reported in the supplementary (Figure S1) [24,25,31,32].

## 4. Discussion

This study provides further evidence regarding the metabolic effects of regular “Navelina” orange consumption in patients with MASLD. The high adherence observed in both groups (96.8% in the experimental arm and 93.1% in the control arm) indicates good compliance with the intervention.

The consumption of whole “Navelina” oranges is of particular interest because it provides a complex food matrix containing dietary fiber, vitamins, carotenoids, flavonoids, and other phytochemicals. Previous evidence indicates that the total polyphenol content of “Navelina” oranges is higher in the flesh tissue than in the juice [17]. In addition, the albedo, the white tissue surrounding the pulp, is particularly rich in flavonoids such as hesperidin, which has been associated with antioxidant and anti-inflammatory properties. Therefore, consumption of the whole fruit may provide a different exposure to bioactive compounds compared with orange juice or isolated citrus extracts.

Against this nutritional background, the present intervention resulted in significant changes in selected nutritional biomarkers. Significant time-by-treatment interactions were observed for dietary sugar, vitamin A, and vitamin C intake indicating different longitudinal changes between the two groups. The increase in dietary vitamin C and vitamin A is consistent with the nutritional contribution of regular orange consumption and confirms that the intervention was able to modify selected nutritional biomarkers. However, these changes should not be interpreted as evidence of clinically relevant improvements in metabolic health.

Of particular interest are the glucose-related findings. Although the time-by-treatment interaction for fructosamine did not reach conventional statistical significance (0.05), the direction of the effect may warrant further investigation. Fructosamine provides an estimate of glycemic exposure over the preceding 2–3 weeks and may therefore provide complementary information in short-term nutritional interventions. However, as fructosamine reflects glycation of serum proteins, mainly albumin, its concentration is also influenced by serum albumin levels and protein turnover, which may be altered in MASLD independently of glycemia.

Previous clinical studies investigating citrus-derived compounds and orange juice have reported mixed findings regarding glucose metabolism. While some studies have reported improvements in specific indices of insulin resistance, others have found no significant overall effects on fasting glucose, insulin levels, or related metabolic markers [33,34]. These differences may reflect variations in study populations, intervention protocols, duration, and the composition of the citrus products investigated.

Previous randomized trials and meta-analyses of orange juice consumption have reported inconsistent effects on glucose, insulin, and HOMA-IR, with some studies suggesting improvements and others finding no significant changes. These discrepancies may reflect differences in intervention duration, dose, participant characteristics, comparator, and the form of citrus consumed. In particular, differences between whole-fruit and juice consumption may be relevant because the food matrix, dietary fiber, and distribution of polyphenols differ between these forms [34]. Thus, the present fructosamine finding should be considered hypothesis-generating rather than confirmatory.

The absence of significant changes in fasting glucose, insulin, HOMA-IR, and QUICKI further suggests that four weeks of whole-orange consumption did not substantially modify conventional fasting measures of glucose homeostasis. This may indicate that any metabolic effects of the intervention are modest or may require longer exposure to become detectable. Importantly, fructosamine provided complementary information on short-term glycemic exposure and showed a decreasing trend in the intervention group (*p* = 0.078); however, this change did not reach statistical significance and, considering that albumin was not measured, should not be interpreted as evidence of improved glycemic control.

The exploratory correlation analyses revealed associations between changes in LDL and indices of insulin resistance in the intervention group. Changes in LDL were positively correlated with HOMA-IR and insulin and inversely correlated with QUICKI, with the strongest association observed between LDL and QUICKI (r = −0.639, *p* < 0.001). These findings are biologically plausible given the close relationship between lipid metabolism and insulin sensitivity. Previous evidence suggests that polyphenols may modulate lipid metabolism through multiple interconnected mechanisms, including effects on hepatic cholesterol synthesis, fatty acid utilization, and oxidative and inflammatory pathways [35,36,37]. However, the present correlations do not demonstrate an intervention-mediated metabolic mechanism. In particular, correlations calculated separately within treatment groups cannot establish treatment specificity without a formal statistical comparison of correlation coefficients. Therefore, these associations should be regarded as exploratory and hypothesis-generating.

The post hoc power analysis for fasting glucose and fructosamine provides additional information on the magnitude of between-group changes. These preliminary estimates will be instrumental in determining the required sample size to achieve statistical significance in a future, adequately powered two-arm randomized clinical trial. However, because these analyses were performed post hoc, they should be interpreted as exploratory and should not be used to redefine the primary findings.

Finally, several limitations should be considered when interpreting these results. The relatively small sample size and the short, four-week duration of the intervention limit the generalizability of the findings and preclude drawing strong causal conclusions. Furthermore, the exploratory nature of the study and the absence of a formal multiplicity strategy for the multiple correlation analyses necessitate a cautious interpretation of statistically significant associations. Dietary intake and adherence were assessed using self-completed diaries and questionnaires, supported by visual guides for portion sizes; however, the possibility of recall and reporting bias cannot be ruled out. In addition, although participants were asked to avoid other citrus fruits and comparable foods, their actual polyphenol intake, as well as circulating or urinary concentrations of citrus-derived metabolites, was not quantified. Moreover, serum albumin and total protein were not measured, and fructosamine concentrations may be influenced by albumin levels and protein turnover, which can be altered in MASLD. In the previously reported analysis of this trial [17], baseline AST and ALT were within the normal range in both arms and did not change significantly after the intervention, while body weight remained stable, arguing against overt hepatocellular injury or marked metabolic changes induced by the intervention. However, transaminases do not directly reflect hepatic synthetic capacity or protein turnover, so this evidence is only indirect and a residual influence on fructosamine cannot be excluded. Fructosamine findings should therefore be confirmed in future studies including albumin-adjusted fructosamine, glycated albumin, HbA1c or continuous glucose monitoring.

Overall, four weeks of consuming 400 g/day of whole “Navelina” oranges produced changes in selected nutritional biomarkers and showed a suggestive, although not statistically significant, effect on fructosamine. The observed exploratory associations between LDL and insulin-sensitivity markers warrant further investigation of the relationship between lipid and glucose metabolism in response to whole citrus fruit consumption. The polyphenol-rich composition of the edible tissues of “Navelina” oranges, particularly the presence of flavonoids such as hesperidin, provides a plausible biological basis for further investigation.

## 5. Conclusions

Taken together, these findings suggest that regular consumption of whole “Navelina” oranges may influence selected metabolic and nutritional parameters in patients with MASLD. To our knowledge, this study adds clinical evidence on the metabolic effects of whole “Navelina” orange consumption in patients with MASLD, with particular interest in the potential contribution of its polyphenol content. The complex nutritional matrix of the whole fruit, including polyphenols and other bioactive compounds, provides a plausible basis for these potential effects and supports further investigation into the potential health effects of whole-orange consumption. However, given the exploratory nature of the study, the short intervention period, and the lack of direct assessment of polyphenol exposure and citrus-derived metabolites, these findings should be considered preliminary.

Further well-powered, long-term randomized controlled trials are needed to clarify the contribution of citrus polyphenols and other bioactive compounds to the metabolic effects of consuming whole oranges in patients with MASLD. This should include their potential relationship with serum lipidomic profiles and markers of inflammation and oxidative stress. Future studies should also include albumin-adjusted fructosamine or complementary markers of glycemic exposure, and investigate dose–response relationships to better characterize the metabolic effects of orange consumption.

## Figures and Tables

**Figure 1:**
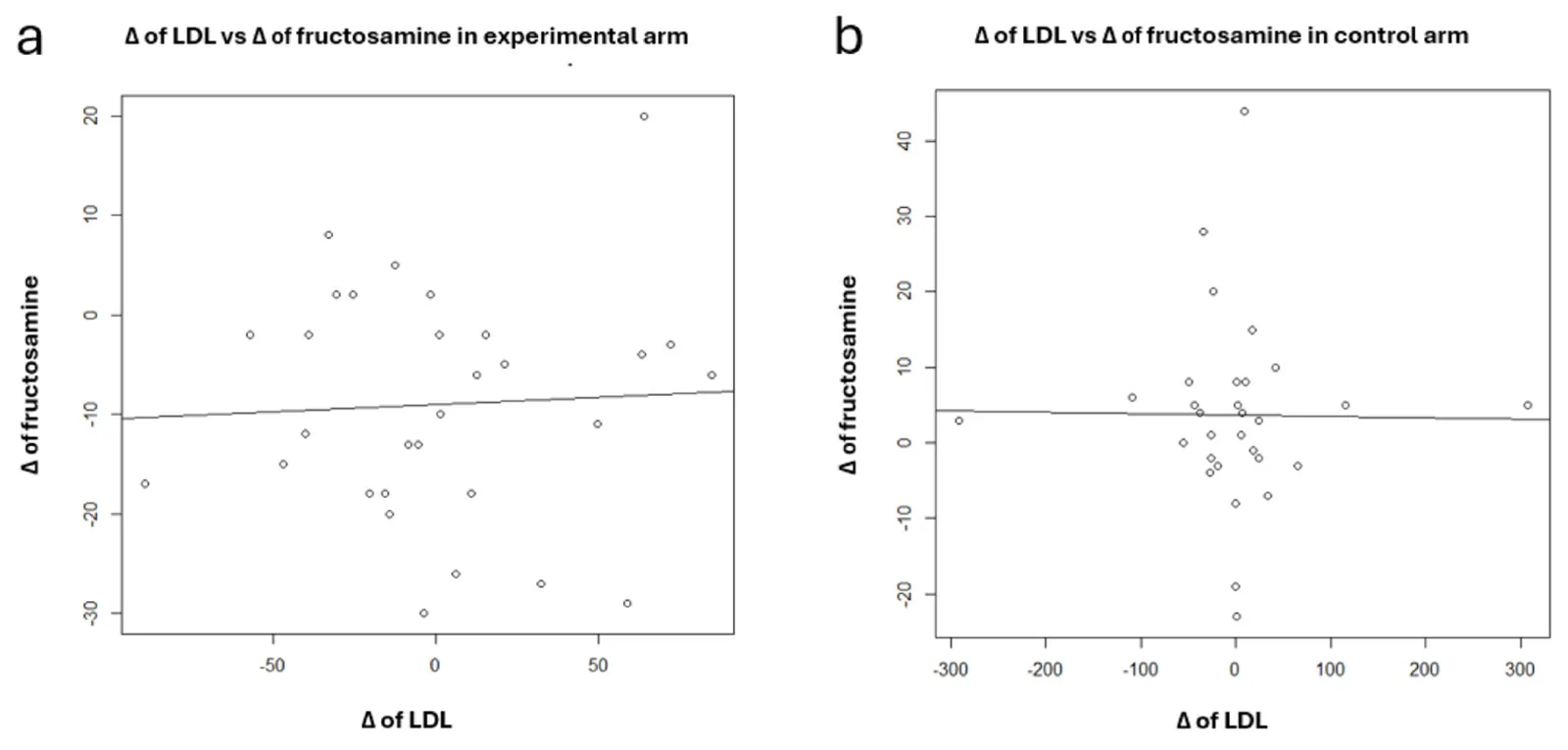

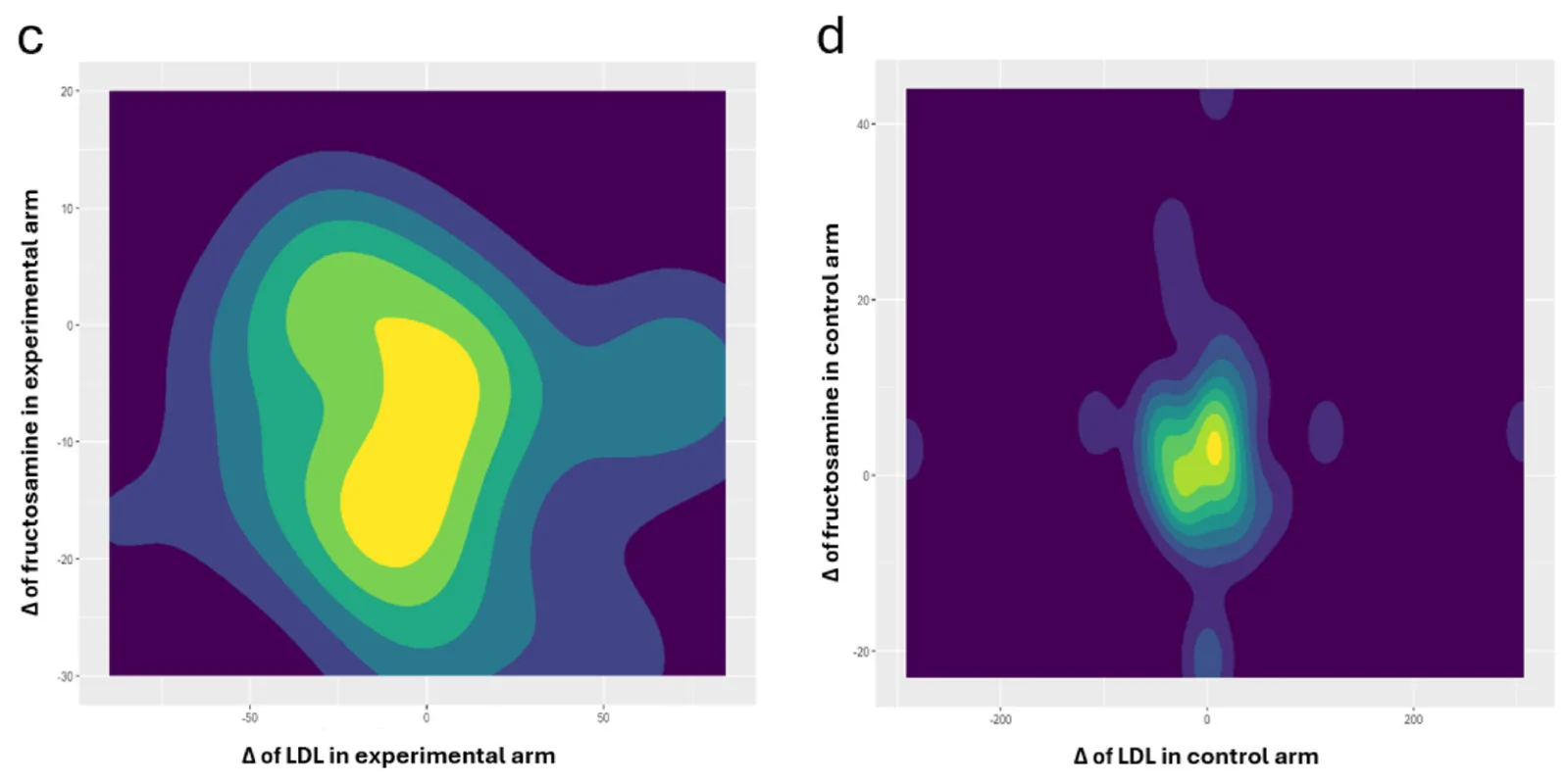
Scatter plots: joint distributions and regression lines of Δ-changes in LDL cholesterol (*x*-axis) and fructosamine (*y*-axis) within the experimental treatment arm (**a**) and control arm (**b**). Contour plots: joint distributions of Δ-changes in LDL cholesterol (*x*-axis) and fructosamine (*y*-axis) within the experimental treatment arm (**c**) and control arm (**d**).

**Figure 2:**
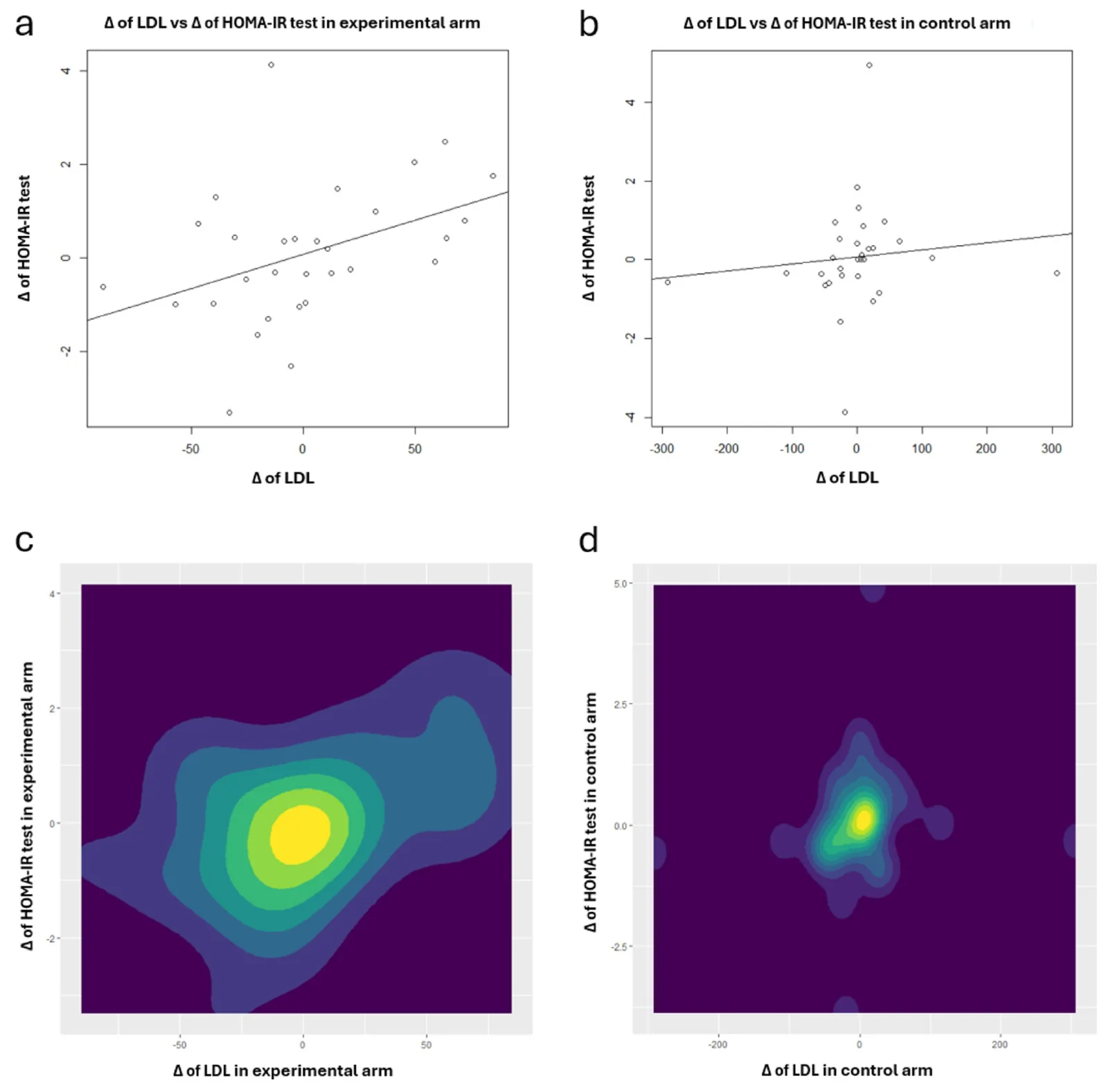
Scatter plots: joint distributions and regression lines of Δ-changes in LDL cholesterol (*x*-axis) and HOMA test (*y*-axis) within the experimental treatment arm (**a**) and control arm (**b**). Contour plots: joint distributions of Δ-changes in LDL cholesterol (*x*-axis) and HOMA-IR test (*y*-axis) within the experimental treatment arm (**c**) and control arm (**d**).

**Figure 3:**
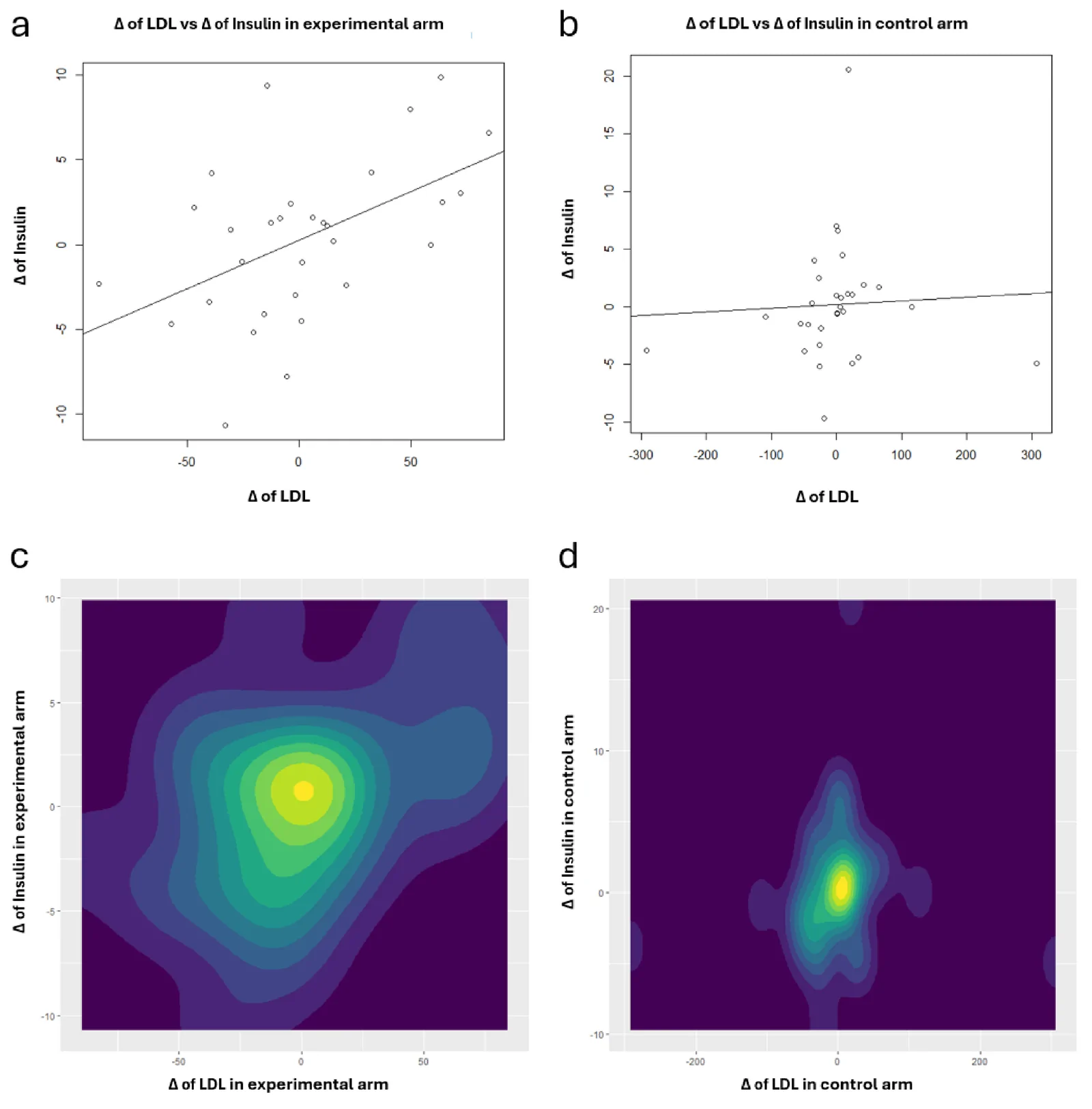
Scatter plots: joint distributions and regression lines of Δ-changes in LDL cholesterol (*x*-axis) and insulin (*y*-axis) within the experimental treatment arm (**a**) and control arm (**b**). Contour plots: joint distributions of Δ-changes in LDL cholesterol (*x*-axis) and insulin (*y*-axis) within the experimental treatment arm (**c**) and control arm (**d**).

**Figure 4:**
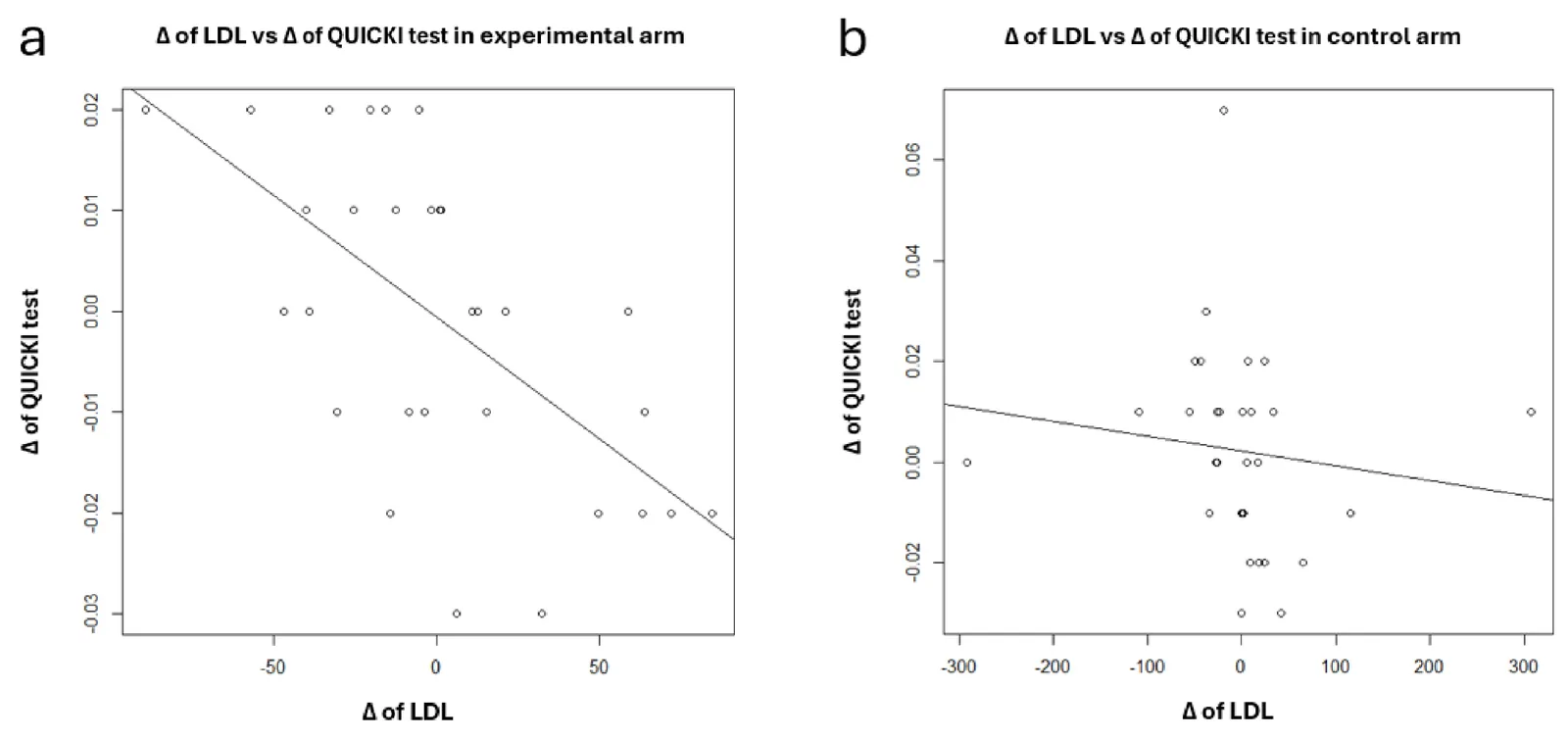

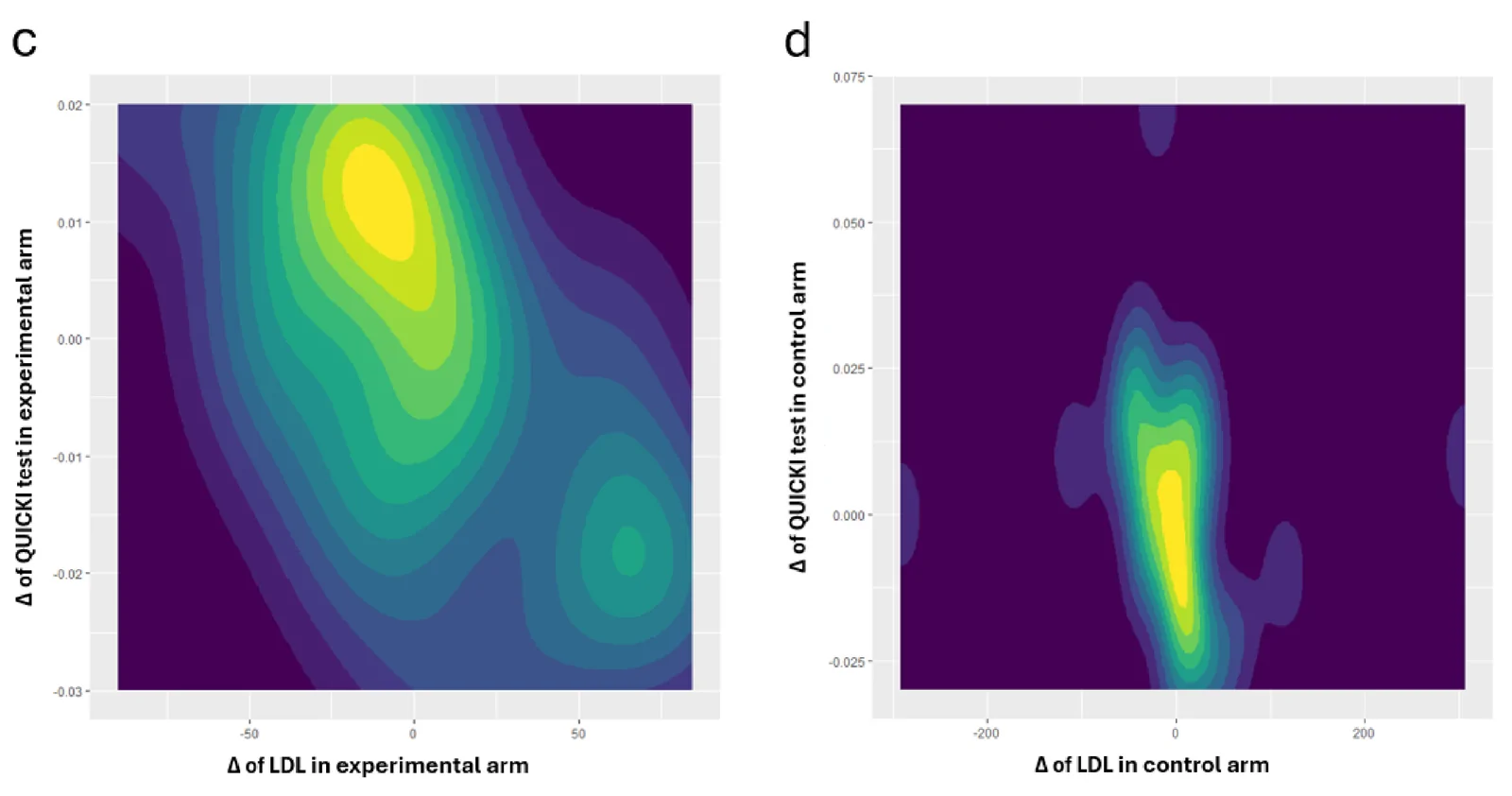
Scatter plots: joint distributions and regression lines of Δ-changes in LDL cholesterol (*x*-axis) and QUICKI test (*y*-axis) within the experimental treatment arm (**a**) and control arm (**b**). Contour plots: joint distributions of Δ-changes in LDL cholesterol (*x*-axis) and QUICKI test (*y*-axis) within the experimental treatment arm (**c**) and control arm (**d**).

**Table 1:** Baseline characteristics of the sample.

Variable	Baseline		
	Experimental Arm (n = 30)	Control Arm (n = 30)	
	*n (%)* or Median [*IQR*]	*n (%)* or Median [*IQR*]	*p*-Value
*Demographic and lifestyle parameters*			
Sex (M/F)	23/7 (76.77/23.33)	20/10 (66.67/33.33)	0.567
Age (years)	54.50 (44.25; 60.75)	52.00 (44.50; 58.50)	0.584
*Anthropometric and clinical parameters*			
Weight (kg)	91.90 (85.25; 97.88)	89.30 (85.98; 99.50)	0.717
BMI (kg/m^2^)	31.55 (29.35; 35.30)	31.10 (28.75; 35.10)	0.679
*Glucose metabolism*			
Fasting blood glucose (mg/dL)	96.50 (91.21; 100.80)	94.00 (88.23; 100.02)	0.200
Fasting insulin (μUI/mL)	16.90 (10.24; 19.20)	12.90 (10.15; 17.48)	0.400
HOMA-IR Test (u)	3.85 (2.39; 4.77)	2.67 (2.29; 4.22)	0.300
QUICKI Test (u)	0.31 (0.30; 0.34)	0.33 (0.31; 0.34)	0.300
Fructosamine (µmol/L)	257.00 (244.05; 266.10)	244.45 (235.09; 258.04)	**0.010**
*Day Dietary Intake*			
Proteins (g)	93.61 (80.99; 106.96)	87.74 (75.53; 94.48)	*0.069*
Lipids (g)	94.46 (84.80; 108.29)	85.39 (76.26; 93.48)	**0.044**
Carbohydrates (g)	194.40 (177.09; 246.07)	237.80 (209.83; 248.18)	*0.062*
Energy intake (Kcal)	2074.73 (1887.17; 2189.95)	1969.62 (1879.47; 2090.30)	0.283
Starch (g)	86.50 (70.50; 104.22)	97.54 (78.25; 109.95)	0.100
Sugars (g)	48.81 (33.79; 60.62)	59.80 (40.68; 76.55)	*0.080*
Total Fiber (g)	10.37 (8.54; 14.99)	11.27 (9.14; 17.39)	0.200
Sodium (mg)	556.06 (356.01; 737.45)	802.45 (584.05; 1030.00)	**0.020**
Potassium (mg)	1159.00 (802.29; 1504.25)	1442.04 (678.09; 1915.15)	0.500
Iron (mg)	6.33 (5.50; 8.18)	7.09 (5.88; 8.85)	0.600
Calcium (mg)	369.08 (277.36; 499.45)	492.25 (373.36; 639.07)	**0.020**
Phosphorus (mg)	741.15 (626.09; 890.01)	793.25 (657.36; 909.61)	0.600
Vitamin B1 (mg)	0.41 (0.35; 0.61)	0.50 (0.32; 0.64)	0.500
Vitamin B2 (mg)	0.65 (0.44; 0.75)	0.71 (0.44; 0.91)	0.500
Vitamin B3 (mg)	9.03 (7.19; 11.23)	8.74 (7.20; 11.04)	1.000
Vitamin A (µg)	356.25 (205.65; 706.14)	533.48 (275.25; 528.78)	0.300
Vitamin C (mg)	33.95 (14.82; 85.95)	45.24 (32.64; 78.61)	0.300
Vitamin E (mg)	7.07 (6.05; 8.95)	6.67 (5.72; 9.02)	0.500
Water (mL)	631.44 (527.05; 780.09)	605.35 (527.44; 748.12)	1.000
Dietary SFAs (g)	34.60 (31.80; 36.51)	33.31 (31.80; 35.43)	0.500
Dietary MUFAs (g)	37.91 (34.91; 40.15)	36.63 (34.90; 38.84)	0.500
Dietary PUFAs (g)	29.63 (27.20; 31.31)	28.62 (27.20; 30.35)	0.500

**Note.** Data are median [*Interquartile range, IQR*]. The *p*-values by Mann–Whitney test or Chi-square. In **bold**, the significant results (*p* < 0.05); in *italics*, trends (0.05 < *p* < 0.10). **Abbreviations:** BMI, Body Mass Index; HOMA-IR test, Homeostatic Model Assessment for Insulin Resistance Test; QUICKI test, Quantitative Insulin Sensitivity Index; SFAs, Saturated Fatty Acids; MUFA, Monounsaturated Fatty Acids; PUFA, Polyunsaturated Fatty Acids.

**Table 2:** Result of the Generalized Estimating Equation (GEE) models.

Outcome	Experimental Arm		Control Arm		GEE		
	Baseline Median (IQR)	Follow Up Median (IQR)	Baseline Median (IQR)	Follow Up Median (IQR)	β_T_ *p*-Value 95%CI	β_TR_ *p*-Value 95%CI	β_TxTR_ *p*-Value 95%CI
***Glucose metabolism***							
Fasting blood glucose (mg/dL)	96.50 (91.20; 100.80)	94.00 (89.23; 99.81)	94.00 (88.23; 100.02)	95.04 (91.13; 100.84)	1.169 0.624 −3.500; 5.838	**8.142** **0.044** **0.229; 16.054**	−2.671 0.650 −14.216; 8.874
Fasting insulin (μUI/mL)	16.90 (10.24; 19.20)	14.80 (10.04; 19.85)	12.90 (10.15; 17.48)	13 (9.30; 17.45)	0.150 0.917 −2.690; 2.991	1.503 0.405 −2.035; 5.041	−0.601 0.797 −5.194; 3.992
HOMA-IR Test (u)	3.85 (2.39; 4.77)	3.47 (2.44; 4.85)	2.67 (2.29; 4.22)	2.90 (2.16; 4.08)	0.063 * 0.897 −0.903; 1.031	0.914 * 0.104 −0.191; 2.020	0.035 * 0.965 −1.528; 1.598
QUICKI Test (u)	0.31 (0.30; 0.34)	0.32 (0.30; 0.33)	0.33 (0.31; 0.34)	0.33 (0.31; 0.35)	0.003 0.674 −0.009; 0.015	−0.009 0.191 −0.023; 0.004	−0.004 0.669 −0.021; 0.014
Fructosamine (μmol/L)	257.00 (244.05; 266.10)	246.08 (238.96; 254.07)	244.45 (235.09; 258.04)	246.32 (237.35; 257.40)	3.607 0.445 −5.6507; 12.865	**13.914** **0.007** **3.772; 24.057**	*−12.638* *0.078* *−26.715; 1.438*
***Dietary intake***							
Proteins (g/day)	93.61 (80.99; 106.96)	92.84 (83.41; 103.64)	87.74 (75.53; 94.48)	90.00 (78.10; 98.92)	4.478 0.228 −2.799; 11.757	5.645 0.138 −1.818; 13.109	−5.698 0.264 −15.705; 4.308
Lipids (g/day)	94.46 (84.80; 108.29)	92.62 (84.71; 100.74)	85.39 (76.26; 93.48)	83.14 (71.89; 93.48)	−3.547 0.362 −11.175; 4.081	2.853 0.523 −5.912; 11.620	1.899 0.729 −8.881; 12.681
Carbohydrates (g/day)	194.40 (177.09; 246.07)	216.15 (196.32; 247.18)	237.80 (209.83; 248.18)	236.36 (209.42; 252.94)	−0.791 0.922 −16.683; 15.102	−14.403 0.190 −35.920; 7.114	11.013 0.424 −15.959; 37.984
Energy intake (Kcal/day)	2074.73 (1887.17; 2189.95)	1988.22 (1878.00; 2233.27)	1969.62 (1879.43; 2090.30)	1981.12 (1865.25; 2095.36)	−12.721 0.513 −50.832; 25.390	14.311 0.417 −20.283; 48.906	18.334 0.636 −57.805; 94.472
Starch (g/day)	86.50 (70.50; 104.22)	87.50 (69.00; 101.92)	97.54 (78.25; 109.95)	93.69 (80.74; 110.02)	3.839 0.53 −8.025; 15.703	−3.755 0.58 −17.117; 9.607	−3.312 0.69 −19.620; 12.994
Sugars (g/day)	48.81 (33.79; 60.62)	74.81 (60.79; 87.32)	59.80 (40.68; 76.55)	62.91 (44.26; 71.54)	1.849 * 0.732 −16.331; 3.200	−6.565 * 0.187 −8.747; 12.445	20.742 * **0.002** **7.427; 34.058**
Total Fiber (g/day)	10.37 (8.54; 14.99)	14.25 (11.52; 18.07)	11.27 (9.14; 17.39)	16.50 (10.39; 20.38)	2.278 * 0.40 −0.612; 5.169	−1.128 * 0.12 −3.781; 1.525	0.890 * 0.61 −2.571; 4.351
Sodium (mg/day)	556.06 (356.01; 737.45)	535.27 (397.93; 866.32)	802.45 (584.05; 1030.00)	712.12 (415.16; 834.43)	−188.938 * 0.319 −560.736; 182.861	−430.88 * **0.008** **−752.041; −109.723**	214.233 * 0.298 −189.689; 618.155
Potassium (mg/day)	1159.00 (802.29; 1504.25)	1685.34 (1415.39; 2220.40)	1442.04 (678.09; 1915.15)	1478.25 (1039.36; 1734.48)	185.24 0.173 −81.144; 451.6203	−199.13 0.152 −471.542; 73.279	*329.37* *0.053* *−3.635; 662.369*
Iron (mg/day)	6.33 (5.50; 8.18)	7.12 (4.76; 9.13)	7.09 (5.88; 8.85)	6.92 (6.20; 8.41)	0.287 0.655 −0.976; 1.552	−0.848 0.211 −2.179; 0.482	−0.233 0.796 −2.004; 1.537
Calcium (mg/day)	369.08 (277.36; 499.45)	586.09 (491.34; 789.43)	492.25 (373.36; 639.07)	522.15 (430.48; 666.63)	77.440 * 0.297 −67.975; 222.86	−75.385 * 0.129 −172.60; 21.83	104.535 * 0.204 −56.713; 265.78
Phosphorus (mg/day)	741.15 (626.09; 890.01)	768.22 (665.18; 1000.00)	793.25 (657.36; 909.61)	784.18 (708.38; 903.43)	70.562 0.27 −55.560; 196.684	−66.236 0.12 −149.236; 16.764	−1.574 0.98 −146.447; 143.299
Vitamin B1 (mg/day)	0.41 (0.35; 0.61)	0.68 (0.55; 0.87)	0.50 (0.32; 0.64)	0.55 (0.44; 0.75)	0.083 0.117 −0.020; 0.187	−0.072 0.190 −0.180; 0.035	*0.137* *0.058* *−0.004; 0.279*
Vitamin B2 (mg/day)	0.65 (0.44; 0.75)	0.82 (0.64; 1.17)	0.71 (0.44; 0.91)	0.62 (0.51; 0.83)	0.032 0.69 −0.122; 0.186	−0.102 0.18 −0.252; 0.046	0.154 0.11 −0.035; 0.345
Vitamin B3 (mg/day)	9.03 (7.19; 11.23)	10.56 (7.52; 12.42)	8.74 (7.20; 11.04)	9.47 (7.21; 13.15)	1.116 0.286 −0.935; 3.169	−0.674 0.391 −2.218; 0.868	−0.244 0.848 −2.755; 2.267
Vitamin A (µg/day)	356.25 (205.65; 706.14)	619.38 (502.36; 778.08)	533.48 (275.25; 528.79)	585.44 (265.05; 794.36)	33.970 0.600 −93.063; 161.004	−72.903 0.228 −191.362; 45.55	**206.521** **0.012** **45.578; 367.464**
Vitamin C (mg/day)	33.95 (14.82; 85.95)	221.70 (207.41; 239.74)	45.24 (32.64; 78.61)	47.73 (34.73; 64.74)	−4.052 * 0.81 −36.622; 28.51	−9.029 * 0.64 −47.208; 29.149	180.835 * **<0.001** **133.166; 228.51**
Vitamin E (mg/day)	7.07 (6.05; 8.95)	5.89 (5.38; 8.23)	6.67 (5.72; 9.02)	5.79 (4.60; 6.33)	−0.476 * 0.607 −2.287; 1.335	−0.198 * 0.714 −1.264; 0.866	−1.104 * 0.313 −3.251; 1.043
Water (mL/day)	631.44 (527.05; 780.09)	774.13 (590.25; 941.01)	605.35 (527.44; 748.12)	718.96 (576.14; 834.34)	**130.977** **0.045** **2.349; 259.61**	−10.743 0.843 −117.387; 95.901	−10.867 0.889 −163.47; 141.74
Dietary SFAs (g/day)	34.60 (31.80; 36.51)	33.80 (32.22; 38.44)	33.31 (31.80; 35.43)	34.15 (32.14; 36.19)	0.242 0.567 −0.588; 1.072	−0.249 0.723 −1.628; 1.129	0.434 0.668 −1.558; 2.427
Dietary MUFAs (g/day)	37.91 (34.91; 40.15)	37.16 (35.34; 42.28)	36.63 (34.90; 38.84)	37.41 (35.37; 39.64)	0.267 0.565 −0.644; 1.179	−0.271 0.724 −1.785; 1.242	0.476 0.669 −1.711; 2.664
Dietary PUFAs (g/day)	29.63 (27.20; 31.31)	29.08 (27.62; 33.00)	28.62 (27.20; 30.35)	29.21 (27.57; 30.89)	0.206 0.569 −0.505; 0.9181	−0.214 0.722 −1.396; 0.968	0.375 0.666 −1.332: 2.083

**Note.** Data are the median [*Interquartile range, IQR]*. GEE: Generalized Estimating Equation (Gamma distribution, link = *identity*). The models’ time parameters (β_T_) are interpreted as average time changes in the outcomes between the two separate measurements (follow up − baseline measurements), the parameters associated with the treatment (β_TR_) are interpreted as average baseline differences between the two treatments (the control one represents the reference category). The interaction parameters (β_TxTR_) are interpreted as average time differences in the outcomes (from baseline) between treatments. GEE models are adjusted for baseline values of lipids, total cholesterol, carbohydrates, proteins and HDL cholesterol; modelling on the covariates (as response variables) causes the relative adjustment to vanish. 95%CI: 95% confidence interval. In **bold**, the significant results (*p* < 0.05); in *italics*, trends (0.05 < *p* < 0.10). * Results achieved by Gaussian GEE. Abbreviations: SFAs, Saturated Fatty Acids; MUFAs, Monounsaturated Fatty Acids; PUFAs, Polyunsaturated Fatty Acids.

**Table 3:** Δ-changes in Spearman correlations between selected glucose metabolism markers and LDL, stratified by arm.

r (*p*-Value)				
**Experimental Arm**	HOMA-IR test	Insulin	QUICKI test	Fructosamine
LDL	**0.471** **(0.009)**	**0.475** **(0.008)**	**−0.639** **(<0.001)**	−0.046 (0.808)
HOMA-IR test	1	**0.931** **(<0.001)**	**−0.886** **(<0.001)**	−0.104 (0.582)
Insulin	-	1	**−0.864** **(<0.001)**	−0.161 (0.395)
QUICKI test	-	-	1	0.154 (0.415)
Fructosamine	-	-	-	1
				
**Control arm**	HOMA-IR test	Insulin	QUICKI test	Fructosamine
LDL	0.290 (0.120)	0.215 (0.252)	*−0.317* *(0.087)*	−0.015 (0.936)
HOMA-IR test	1	**0.961** **(<0.001)**	**−0.764** **(<0.001)**	0.108 (0.569)
Insulin	-	1	**−0.731** **(<0.001)**	0.069 (0.715)
QUICKI test	-	-	1	0.005 (0.979)
Fructosamine	-	-	-	1

**Note.** r: Spearman correlation. In **bold**, the significant results (*p*-value < 0.05); in *italics*, trends (0.05 < *p*-values < 0.10). **Abbreviations**: LDL, Low-Density Lipoprotein; HOMA-IR test, Homeostatic Model Assessment for Insulin Resistance; QUICKI test, Quantitative Insulin Sensitivity Index.

## Data Availability

Data are available from corresponding author upon reasonable requests.

## Supplementary Materials

The following supporting information can be downloaded at https://www.mdpi.com/article/10.3390/nu18213668/s1: Figure S1: Post hoc power analysis for the effects of time and treatment on fasting blood glucose and fructosamine levels.

## Abbreviations

The following abbreviations are used in this manuscript:

MASLD	Metabolic Dysfunction-Associated Steatotic Liver Disease
HOMA-IR	homeostatic model assessment for insulin resistance
QUICKI	quantitative insulin sensitivity check index
AGE	altered advanced glycation end-products
sRAGE	receptor for altered advanced glycation end-products
HDL	high-density lipoprotein
EPA	eicosapentaenoic acid
PUFAs	polyunsaturated fatty acids
MUFAs	monounsaturated fatty acids
AA	arachidonic acid
SFAs	saturated fatty acids
IQR	Interquartile range
GEE	Generalized Estimating Equation
SMD	Standardized Mean Difference
CAP	Controlled Attenuation Parameter
BMI	Body Mass Index
LDL	Low-Density Lipoprotein
