# Supplementary material for: Effects of Whole “Navelina” Orange Consumption on Glucose Homeostasis and Fructosamine Levels in Patients with Metabolic Dysfunction-Associated Steatotic Liver Disease (MASLD): A Randomized Controlled Trial
Source: Nutrients. 2026 Nov 5;18(21):3668. doi: 10.3390/nu18213668 (PMC13766203; doi:10.3390/nu18213668)
Supplement: Supplementary file 1 [file nutrients-18-03668-s001.zip › nutrients-4640407-supplementary.pdf]

Supplementary

Post hoc power analysis

We performed a post hoc power analysis using Cohen's *d* (i.e., the standardized mean difference, SMD, of the delta changes [follow-up − baseline]) as the effect size [24,25] for fasting blood glucose and fructosamine levels. Post hoc power analysis provides an estimate of the statistical power of a test based on the observed effect size and sample size. The rationale underlying this approach is to assess whether a non-significant result may be attributable to insufficient statistical power [31]. To obtain the complete range of eligible sample size values, we further evaluated the performance of the post hoc power analysis through a simulation study, varying the power values (x-axis) and determining the corresponding sample sizes (y-axis) based on the observed effect size. The two-sided Type I error rate was set at 0.05. Nevertheless, it should be noted that post hoc power analysis has been criticized, as extensively discussed by Hoenig and Heisey [32].

Based on these calculations, the post hoc analysis yielded a power of 0.092, corresponding to the observed standardized mean difference in changes (Δ) in fasting blood glucose (SMD = 0.152). Subsequently, the simulation study estimated the sample sizes associated with different power values, thereby allowing comparison with the power achieved in the present study (Figure S1, panel a). For fructosamine, the observed power was 0.672, based on an SMD of 0.631 (Figure S1, panel b).

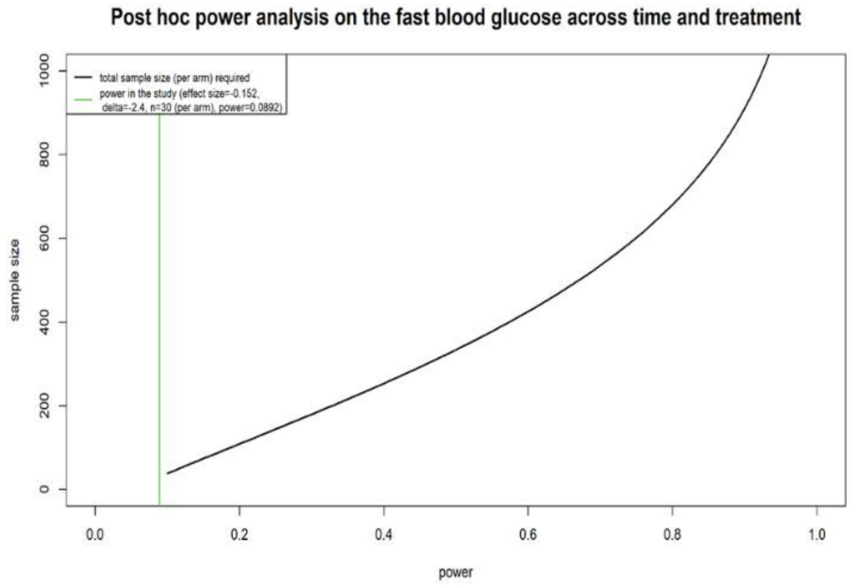

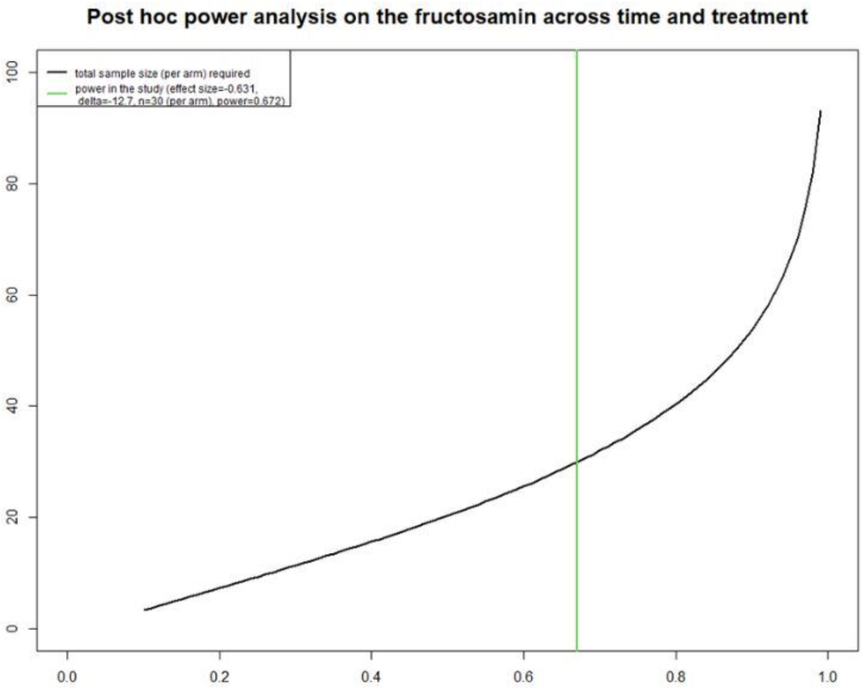

Note. *Δ Experimental treatment Arm – Δ Control Arm

References

[24] Cohen, J. Statistical Power Analysis for the Behavioral Sciences, 2nd ed.; Academic Press: New York, NY, USA, 1988. 525

[25] Cohen, J. Things I have learned (so far). *Am. Psychol.* **1990**, *45*, 1304–1312.

[31] Crespi, C.M. Power and Sample Size in R; Chapman & Hall: Boca Raton, FL, USA, **2020**; ISBN 9781138591622

[32] Hoening, J.M.; Heisey, D.M. The Abuse of Power: The Pervasive Fallacy of Power Calculations for Data Analysis. *Am. Stat.* **2001**, *55*, 19–24.
